# Neurological, cognitive and psychiatric features of Primary Progressive Aphasia: a naturalistic study of a large cohort over 10 years

**DOI:** 10.1002/alz.088276

**Published:** 2025-01-03

**Authors:** Maud Tastevin, Monica Lavoie, Robert Laforce

**Affiliations:** ^1^ Clinique Interdisciplinaire de Mémoire, CHU de Québec‐Université Laval, Quebec, QC Canada; ^2^ Research Chair on Primary Progressive Aphasia ‐ Fondation de la famille Lemaire, Quebec, QC Canada; ^3^ Faculté de Médecine, Université Laval, Quebec, QC Canada; ^4^ Centre de Recherche du CHU de Québec‐Université Laval, Québec, QC Canada

## Abstract

**Background:**

Patients with Primary Progressive Aphasias (PPAs) almost systematically inquire about the longitudinal evolution of their disease in clinics but very little research exists on the issue.

**Method:**

We studied 82 PPA patients from the Research Chair on PPA – Fondation de la Famille Lemaire Cohort over a 10‐year span (42 logopenic, 21 non‐fluent/agrammatic and 19 semantic PPAs) and collected data from 5 domains (language, cognition, motor, psychiatric, functional) at 5 time points from onset to death. Logistical regression analyses and repeated measures ANOVAs were conducted to delineate the longitudinal profile of each variant PPA.

**Result:**

All patients presented anomia and executive impairments over time. Language deficits tended to be more significant for lvPPA, particularly after 3 years of evolution and this group showed broader cognitive impairments. Psychiatric symptoms were more frequent in svPPA and nfvPPA, particularly after 5 years of evolution. Motor features predominantly affected patients with nfvPPA after 2 years of evolution. Overall functional abilities remained preserved the longest in svPPA (up to 5 years).

**Conclusion:**

To our knowledge, this naturalistic study on all major PPA symptoms over a 10‐year span from onset to death is the largest to date. Data from this study can help clinicians better inform and prepare their patients for future challenges as well as design more focused interventions.

